# Sensitivity and Specificity Assessment of Various African Swine Fever ELISA Kits for Accurate Detection of Seropositive Wild Boar

**DOI:** 10.3390/pathogens15040360

**Published:** 2026-03-28

**Authors:** Virginia Friedrichs, Alexander Schäfer, Paul Deutschmann, Sabine Bock, Andreas Hlinak, Wulf-Iwo Bock, Andreas Moss, Martin Beer, Sandra Blome

**Affiliations:** 1 Friedrich-Loeffler-Institut, Suedufer 10, 17493 Greifswald-Insel Riems, Germany; virginia.friedrichs@fli.de (V.F.); alexander.schaefer@fli.de (A.S.); paul.deutschmann@fli.de (P.D.); martin.beer@fli.de (M.B.); 2 Landesuntersuchungsanstalt für das Gesundheits- und Veterinärwesen Sachsen (LUA), Jaegerstrasse 8/10, 01099 Dresden, Germany; 3 Landeslabor Berlin-Brandenburg (LLBB), Rudower Chaussee 39, 12489 Berlin, Germany; sabine.bock@landeslabor-bbb.de (S.B.); wulf-iwo.bock@landeslabor-bbb.de (W.-I.B.); 4 Niedersächsisches Landesamt für Verbraucherschutz und Lebensmittelsicherheit (LAVES), Stau 75, 26122 Oldenburg, Germany; andreas.moss@laves.niedersachsen.de

**Keywords:** African swine fever virus, serology, field samples, wild boar, ELISA, sensitivity, specificity

## Abstract

The continuous and rapid spread of African swine fever (ASF) still poses a significant threat to Eurasian wild boar and domestic pigs, leading to substantial economic losses in all associated sectors annually. In Europe, including Germany, affected wild boar populations have become an important driver and host of ASF virus (ASFV), and monitoring and surveillance is key to tailor control measures that impede viral spread. While molecular methods are used to confirm the disease and to monitor viral evolution, serology gains importance in endemically affected regions as it provides insights into disease dynamics and possible attenuation of ASFV strains. Frontline serological diagnosis is done using ELISA assays, of which several are commercialized. However, accurate performance of ELISA assays is key for correct interpretation of wild boar samples. Due to the various stages of hemolysis and decay, field samples from wild boar can be challenging for ELISA assays. To assess which indirect or competitive ELISA kit performs best when dealing with such samples, we compared the sensitivity and specificity of four commercially available ELISA kits that are licensed in Germany, as well as three unlicensed but commercially available kits. For this comparison, we used all wild boar samples submitted to the National Reference Laboratory (NRL) for ASF in years 2021 and 2022, as well as samples from domestic pigs to have a control cohort where sample quality is optimal. We observed that wild boar samples, varying in stage of hemolysis and decay, were challenging for all kits included in this study. However, samples of domestic pigs were largely interpreted correctly by ELISA, using immunoperoxidase test as verification method. Additionally, the comparability of results obtained by regional laboratories was high. Our study provides data that highlights the importance of careful kit selection, assessment of sample quality, and data interpretation for effective ASFV surveillance and control.

## 1. Introduction

African swine fever (ASF) is a viral disease affecting domestic pigs and wild suid species caused by the ASF virus (ASFV). In domestic pigs and Eurasian wild pigs, such as European wild boar, the notifiable disease manifests by inducing high fever, loss of appetite, and hemorrhages in the skin and multiple organs; lethality rates are exceptionally high. Over the last two decades, ASF has gained unprecedented spatial distribution and is now affecting many regions in the world (World Animal Health Information System, WAHIS, visited 14th September 2025). In Europe, including Germany, affected wild boar populations have become an important driver and reservoir of ASF virus (ASFV) [1]. In the absence of widely licensed vaccines or treatment options, ASF has a significant impact on animal health and associated economies.

Against this background, early detection of infected individuals and assessment of prevalence and outbreak dimensions are essential to install rapid and tailored control measures and prevent further spread of the virus. Due to the rapid clinical progression and high lethality in acutely or peracutely infected animals, humoral antibody responses often fail to develop to detectable levels, rendering serological assays of limited diagnostic value in such cases [2] Conversely, antibody detection assumes greater significance in endemically affected populations and in areas where attenuated virus strains are circulating [3]. In this context, assessment of ASFV genome in qPCR and antibodies in ELISA should be conducted back-to-back to gain a full overview of disease dynamics and possible changes in disease phenotype [4]. Another important application of serology would be surveillance after any large-scale oral vaccination campaign. Here, classical swine fever vaccination could serve as suitable reference [5].

Different ELISA assays are routinely used for the detection of ASFV-specific antibodies in pigs and a growing number of kits have been commercialized and partially licensed, depending on the country’s prerequisites. However, the performance of these tests and therefore accuracy of results can be significantly affected by the quality of the input sample. In particular, field samples of wild boar are worth mentioning, since these samples often vary in the degree of hemolysis and decay as they are taken from fallen animals or in the framework of hunting actions [1]. Both factors render samples suboptimal, as degraded proteins and other substances can interfere and lead to false results. It is also noteworthy that most manufacturers provide an overview of how a serum/plasma sample should be taken and stored, to ensure optimal assay performance. Wild boar samples hardly meet these criteria, raising questions toward assay validity, especially in high-impact cases (legal outbreak confirmation). Aside from the time of detection of a carcass, environmental factors such as season, weather, and decomposer (e.g., beetles, fungi) activity can lead to variations in the decomposition process, even if individuals are of similar size [6,7].

In this study, we explored the performance of seven different commercially available ELISA kits for the detection of ASFV-seropositive domestic pigs and wild boar. As Germany has an official licensing process for any kits applied in the country for notifiable and reportable animal diseases, we focused on four ELISAs that are already licensed in Germany. Moreover, we included three additional kits with full commercialization but without marketing authorization in Germany. Several ASFV proteins are commonly used as ELISA antigens, including the early structural protein p30, the later capsid protein p72, and multi-antigen combinations containing p32 or p54. These antigens differ in expression kinetics and immunogenicity, which may translate into stage-dependent differences in diagnostic sensitivity [8,9].

To address the issue that sample quality may significantly impact the assay performance, we chose a panel of ‘good-quality’ samples of domestic pigs as performance controls (meeting all sampling and storage criteria) and ‘suboptimal-quality’ field samples of wild boar. Wild boar samples that were derived from the diagnostic submissions to the German National Reference Laboratory (NRL) for serological confirmation in 2021 and 2022 varied greatly in their stage of decay and hemolysis, while samples from domestic pigs were freshly prepared and adequately stored at all times. Based on this sample set, we compared the overall sensitivity and specificity of both indirect and competitive assays. The results of this study provide comprehensive insights into the overall performance of routinely used ASFV-specific ELISA kits with a focus on kits that are licensed in Germany. Including assessment of routine diagnosis in affected and unaffected regions of Germany, and a small ring trial for regional laboratories in the German Federal States of Lower Saxony, Saxony and Berlin-Brandenburg, provided the opportunity to assess the comparability of multisite testing and differences in diagnostic workflows (e.g., automatic or manual platforms).

Hence, our study provides a comprehensive overview of the performance of the ELISA kits for ASFV antibodies licensed in Germany, as well as future products for this market. The data provide the basis for kit selection towards a reliable estimation of ASF prevalence and spread in endemic areas (here Saxony and Berlin-Brandenburg), as well as areas still free of ASFV (here Lower Saxony). Since ASF monitoring guidelines indicate that back-to-back testing of genome and antibody (qPCR and ELISA) is required for accurate assessment of the epidemiological status in a specific region, our study provides reliable and comprehensive datasets to choose a suitable kit for the serological part of surveillance.

## 2. Materials and Methods

### 2.1. Specificity and Sensitivity Assessment

All samples used in this study either originate from the field (wild boar cases or outbreaks in domestic pigs) or animal experiments conducted in high-containment facilities at the Friedrich-Loeffler-Institut (FLI). All animal trials were performed in accordance with current regulations for animal welfare in Germany. The trials were approved by the competent authority (Landesamt für Landwirtschaft, Lebensmittelsicherheit und Fischerei Mecklenburg-Vorpommern [LALLF M-V]) under file references 7221.3-1.1-004/20, 7221.3-1-021/15, 7221.3-1-021/15, 7221.3-1.1-038/13, and 7221.3-2-023/15.

Specifically, field samples from wild boar (*n* = 420), samples from domestic pig outbreaks (*n* = 92) and samples from animal trials (*n* = 185, of which *n* = 5 are wild boar and *n* = 180 domestic pig) were included. All wild boar samples were collected in Saxony in 2021 and 2022 and had been sent to the NRL for ASF at the FLI for confirmation after initial testing at the regional laboratory in Saxony. Usually, only samples rendering positive or questionable ELISA results at regional laboratories are sent to the NRL at FLI for confirmation. However, in order to have a representative cohort and avoid pre-selection biases, negative samples were also sent to FLI. The total number of samples from wild boar and domestic pig tested with each ELISA kit, the manufacturer of each kit, as well as antigen specificities are listed in Table 1. All assays were performed according to the manufacturers’ instructions. The indirect and competitive assays by ID.vet and Gold Standard Diagnostics are licensed in Germany and commercially available, while products of Indical, BioStone and Tetracore are still under evaluation in Germany, but already fully commercialized. To be able to determine correct detection and interpretation of each sample, an immunoperoxidase test (IPT) was performed on all samples included in this study. The IPT is to date the most sensitive assay to specify if a serum sample contains ASFV-specific antibodies; all sera were diluted 1:40 in IPT testing, as stated in accredited protocols of the NRL (in compliance with standard procedures of the European Union Reference Laboratory for ASF). To calculate specificity and sensitivity of each assay, the samples were either defined as true negative (TN), true positive (TP), false negative (FN), or false positive (FP), based on IPT results. Samples falling into the manufacturer-defined ‘questionable’ range are included in the Section 3 (e.g., respective cutoffs in figures) for transparency, but were excluded from sensitivity and specificity calculations, since they cannot be unequivocally assigned to TN/FN/TP/FP.

### 2.2. Comparability of Results During Multisite Testing

To assess the performance of ELISA kits during routine testing at accredited regional laboratories, the following kits were distributed to the LAVES (Lower Saxony State Office for Consumer Protection and Food Safety), LLBB (Berlin-Brandenburg State Laboratory), and LUA (State Institute for Health and Veterinary Investigation in Saxony): ID Screen ASF Indirect, ID Screen ASF Competition, Ingezim ASFV-R, Ingezim PPA COMPAC, and AsurDx ASFV Ab Test. All regional laboratories tested the kits with samples from wild boar submitted for serological testing (mainly from unaffected regions; thus, the results do not reflect the seroprevalence in the restriction zones at that time) and the total number of samples tested at each laboratory is listed in Table 2. All samples that were interpreted as positive or questionable during routine testing at regional laboratories were submitted to FLI for validation by IPT. However, due to limitations in sample volume, positive and questionable samples detected at LLBB could not be validated by IPT. Furthermore, we aimed to determine whether results vary if the same panel of samples is evaluated with the same ELISA kits, but at different laboratories. Therefore, a panel including serum samples from wild boar (*n* = 32) and domestic pigs (*n* = 14) was distributed to all participating regional authorities by the FLI. These samples were subjected to testing with the same kits as wild boar field samples.

## 3. Results

### 3.1. Performance Comparison of Various ASFV-Specific ELISA Kits Using Wild Boar and Domestic Pig Samples

**Figure 1:**
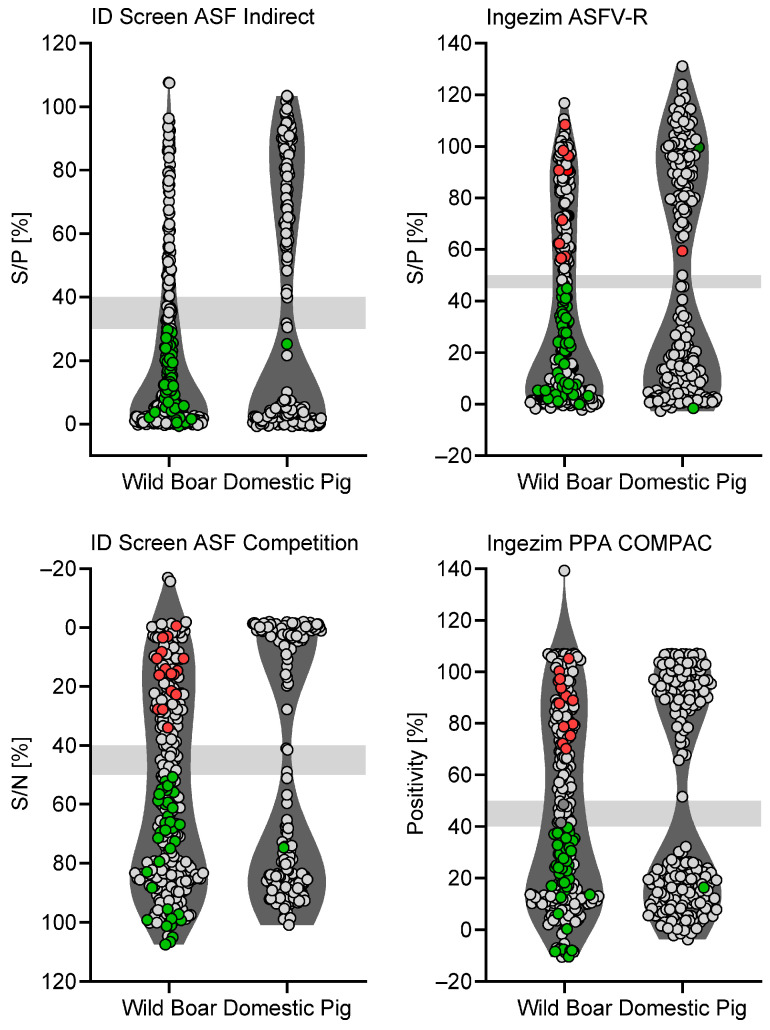
**Comparative testing of licensed ELISA kits.** Field samples from wild boar and domestic pig, as well as samples from animal trials with wild boar and domestic pigs were used. A total of 310 wild boar samples and 229 domestic pig samples were used in this study. Samples were tested with licensed indirect assays (**top row**) and licensed competitive assays (**bottom row**). False-positive results are indicated in red, and false-negative results in green. Points within light gray areas are questionable.

**Figure 2:**
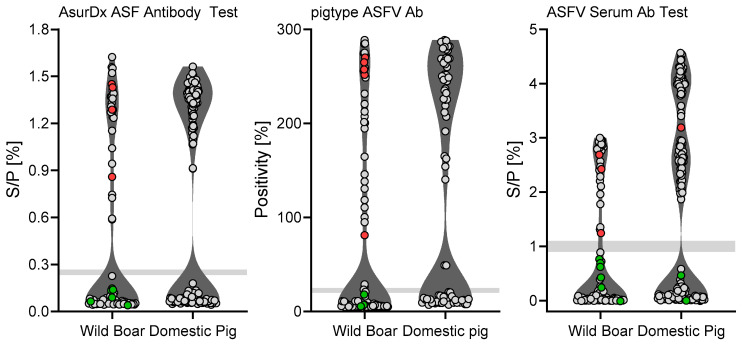
**Comparative testing of not-yet-licensed ELISA kits.** Field samples from wild boar and domestic pig, as well as samples from animal trials with wild boar and domestic pigs were used. A total of 91 wild boar samples and 183 domestic pig samples were used in this study. False-positive results are indicated in red, and false-negative results in green. Points within light gray areas are questionable.

The overall performance of all ELISA kits was assessed by determining the ASFV serological status of samples from wild boar and domestic pigs. Both indirect and competitive assays were able to correctly determine the serological status of all samples from domestic pigs (Figure 1 and Figure 2). Generally, all kits performed more accurately on samples of domestic pigs than field samples of wild boar. The wild boar cohort shows a spread in all graphs while the domestic pig cohort was largely explicitly positive or negative (very few questionable results). Licensed indirect and competitive ELISA assays include results of 310 wild boar and 229 domestic pig samples (Figure 1). It is evident that, independent of the manufacturer, wild boar samples were more diverse compared to distinct results obtained with samples of domestic pig.

Measurements with not-yet-licensed indirect kits included 91 samples originating from wild boar and 183 domestic pig samples (Figure 2). The markedly lower number of wild boar samples tested with these kits, compared to 310 wild boar samples assessed with the licensed assays, results in a smaller and potentially less heterogenous cohort. This is especially relevant for wild boar samples, since they often originate from carcasses. Consequently, the sensitivity and specificity values for the not-yet-licensed kits are associated with broader confidence intervals and a higher risk that their apparent high performance may be overestimated. Direct numerical comparisons between licensed and unlicensed kits, especially wild boar samples, should therefore be interpreted with caution.

**Figure 3:**
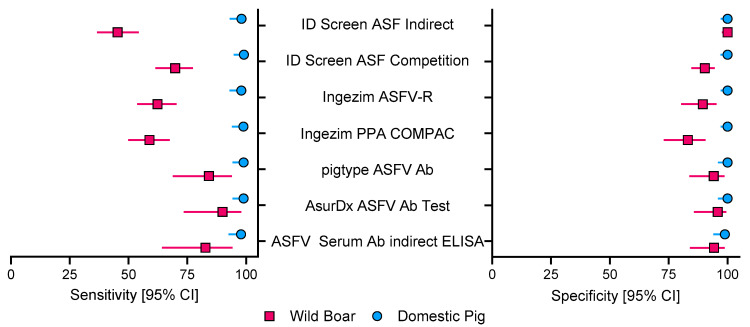
**Sensitivity and specificity evaluation of ELISA kits detecting ASFV-targeting antibodies.** Sensitivity (**left panel)** and specificity (**right panel**) are shown with 95% confidence intervals (CIs).

The allocation of all samples based on IPT results or defined as questionable are stated in Table 3. Samples rendering a questionable result in ELISA were excluded from sensitivity and specificity assessments. Only 1–2 samples out of 229 (1.–4.) or 183 (5.–7.) samples tested were interpreted as false negative, 1–2 were interpreted as questionable, and only one sample was detected as FP by the ASFV Serum Ab indirect ELISA. Taking samples of wild boar and domestic pig together, specificity/sensitivity was 100%/69.26% for the ID Screen ASF Indirect kit, 94.6%/82.2% for the ID Screen ASF Competition kit, 96.73%/77.8% for the Ingezim ASFV-R kit, 95.34%/76.92% for the Ingezim PPA COMPAC kit, 97.28%/94.49% for the pigtype ASFV Ab kit, 95.86%/96.75% for the AsurDx ASFV Ab Test kit, and 97.35%/93.5% for the ASFV Serum Ab indirect ELISA (refer to Figure 3 for confidence interval values for specificity and sensitivity of each kit and pig cohort). However, since wild boar samples were suboptimal due to various stages of decomposition, specificity and sensitivity were also determined separately. For samples of domestic pigs, specificity/sensitivity was 100%/98.02% for the ID Screen ASF Indirect kit, 100%/99.04% for the ID Screen ASF Competition kit, 100%/97.98% for the Ingezim ASFV-R kit, 100%/98.88% for the Ingezim PPA COMPAC kit, 100%/98.94% for the pigtype ASFV Ab kit, 100%/98.94% for the AsurDxASFV Ab Test kit, and 98.89%/97.87% for the ASFV Serum Ab indirect ELISA. For samples of wild boar, specificity/sensitivity was 100%/45.38% for the ID Screen ASF Indirect kit, 90.38%/69.85% for the ID Screen ASF Competition kit, 89.47%/62.32% for the Ingezim ASFV-R kit, 83.12%/58.91% for the Ingezim PPA COMPAC kit, 94.12%/84.21% for the pigtype ASFV Ab kit, 95.83%/90% for the AsurDx ASFV Ab Test kit, and 94.23%/82.76% for the ASFV Serum Ab indirect ELISA. Since variation within a cohort is essential to assess the performance of a kit, the confidence intervals (CIs) for each kit and the corresponding wild boar/domestic pig cohort are shown in Figure 3.

### 3.2. Assessment of Kit Performance During Multisite Testing of Wild Boar Field Samples

**Figure 4:**
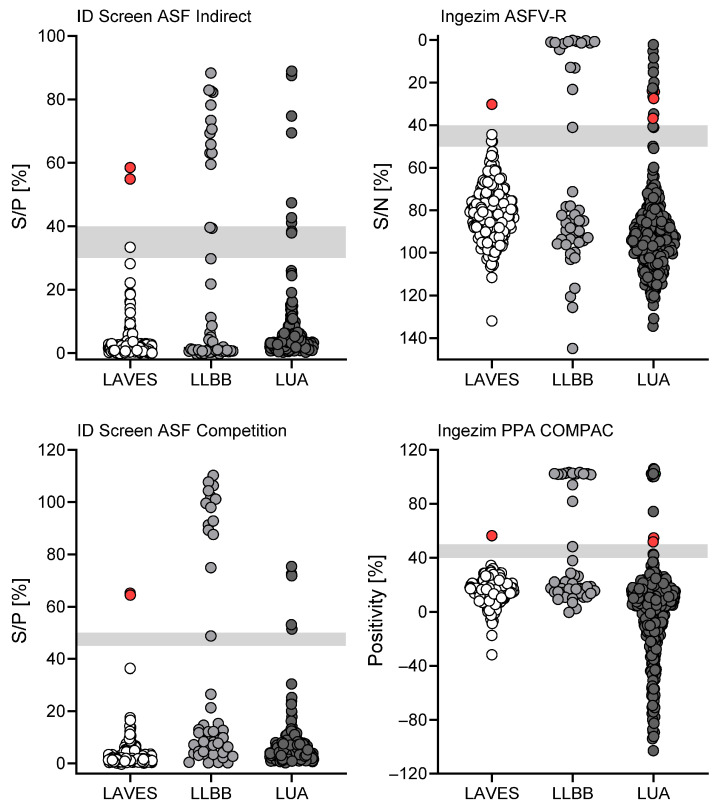
**Performance of all licensed ELISA kits during routine testing at regional laboratories.** Field samples from wild boar were used. A total of 285 wild boar samples were tested at the LAVES (Lower Saxony, white dots), 40 samples were tested at the LLBB (Berlin-Brandenburg, light gray dots), and 368 samples were tested at the LUA (Saxony, dark gray dots). False-positive results are indicated in red. Points within gray areas were questionable.

Since routine diagnostic testing and confirmation of ASFV samples usually includes only samples that were interpreted as positive or questionable for ASFV antibodies during testing in regional laboratories being sent to the FLI for validation, we aimed to assess the performance of all accredited kits for the whole wild boar cohort, not only samples that were sent to FLI (Figure 4). Therefore, all four accredited ELISA kits were distributed to the LAVES (Lower Saxony), the LLBB (Berlin-Brandenburg), and the LUA (Saxony) to test the kit performance during routine testing of samples coming from regions with different epidemiological background. Lower Saxony is still free of ASFV and all positive results obtained at the LAVES turned out to be false positive after validation with IPT. Both licensed indirect assays rendered two false-positive results (panel A), while both competitive assays rendered one false-positive result (panel B). All other samples were interpreted correctly. Samples tested at the LLBB could not be validated due to limited sample volume. The wild boar cohort tested at the LUA rendered no false-positive or false-negative results when tested with the ID Screen ASF Indirect kit, two false-negative results in the Ingezim ASFV-R kit, and four false-positive results each when tested with the competitive kits.

### 3.3. Ring Trial to Evaluate Comparability of Results Obtained at Regional Laboratories Using One Serum Sample Panel

**Figure 5:**
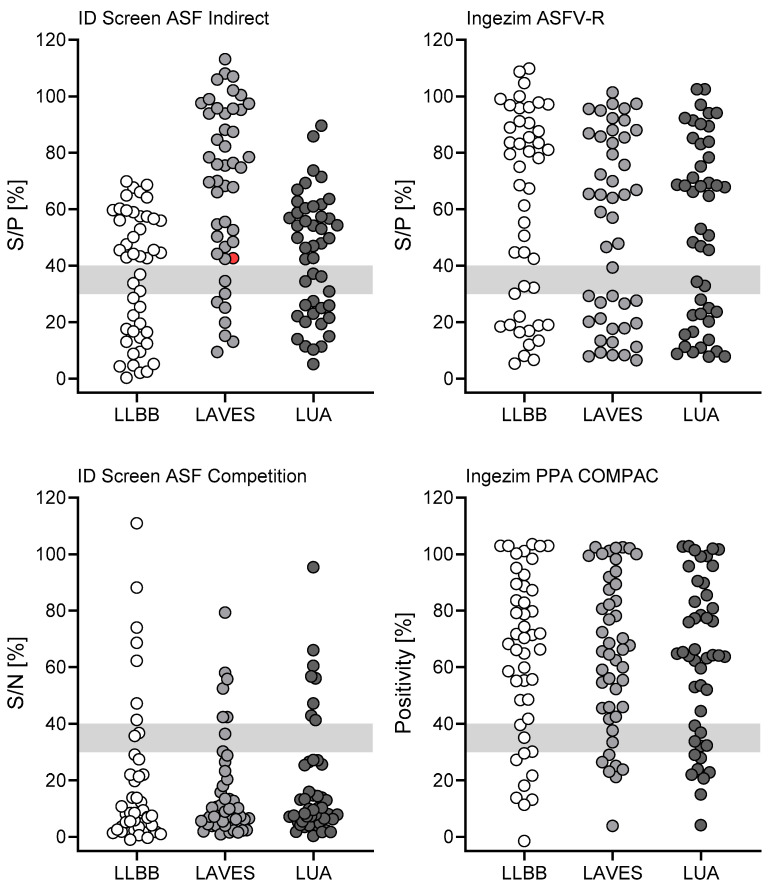
**Ring trial to assess comparability of results obtained at three regional laboratories.** A total of 46 sera were provided by the FLI, including four wild boar sera titrated 1:2, resulting in eight dilutions per serum (32 samples). Additionally, 14 sera differing in positivity and including one true-negative serum were used. All regional laboratories tested this panel in indirect (**top row**) and competitive assays (**bottom row**). A false-positive result is indicated in red. Points within light gray areas were questionable. Samples tested at LLBB are indicated as white dots, at LAVES as light gray dots, and at LUA as dark gray dots.

To exclude the possibility of variations that are introduced by testing at multiple sites and therefore different laboratories, all participating laboratories received a serum panel from the FLI. All participating laboratories are accredited and should therefore generate reproducible results, as validated by proficiency testing conducted at such laboratories. All regional laboratories (LAVES, LLBB, and LUA) received a panel of 46 serum samples from wild boar, encompassing four sera of wild boar with eight 1:2 dilution steps (up to 1:256) and a serum panel of 13 positive serums and one negative serum (Figure 5). Since titrated sera challenge the detection limit of each kit, even high dilutions were not considered false negative even if detected as such. This is due to the fact that, e.g., titration steps 1:32, 1:64, 1:128, and 1:256 of wild boar serum were negative in all laboratories when tested with the ID Screen ASF Indirect kit. Therefore, results among laboratories were comparable, which was the scope of this experiment. However, the one true-negative serum included in the panel was interpreted as positive by LAVES when tested with the ID Screen ASF Indirect kit while it was interpreted as negative by all other laboratories, which is why it was marked as ‘false positive’ (red dot, Figure 5, upper left panel). Overall, the results were largely comparable between laboratories, with only a single clear false-positive result and generally higher S/P% achieved by LAVES when testing the samples with the ID Screen ASF Indirect kit. Follow-up investigations did not provide a sound explanation, especially as this phenomenon was restricted to one test. It is also noteworthy that the laboratory took part in inter-laboratory trials with comparable results in ASF serology. This outlier underscores that, despite good overall agreement, site-specific technical factors should be considered and periodically reviewed, especially when automated platforms are used. However, as all questionable or positive results would be confirmed by the NRL, the impact would have been limited.

## 4. Discussion

With the continued spread of ASF, serology and qPCR are important tools to monitor disease dynamics and the potential evolution and spread of attenuated ASFV strains with reduced mortality and potentially develop vaccination campaigns. An example for the detection of a changing disease phenotype can be found in Estonia. There, an attenuated strain (‘Estonia 2014’) was found in the wild boar population that led to reduced mortality and higher seroprevalence in the hunting bag [6]. Unfortunately, wild boar samples are quite challenging for serology. For this reason, we addressed the performance characteristics of antibody ELISAs using sera of varying quality.

This study provides a comprehensive evaluation of seven commercially available ELISA kits for detecting ASFV antibodies in serum samples from wild boar and domestic pig, with a particular focus on the challenges posed by hemolysis or advanced decomposition of wild boar samples. Four of these tests hold a German license (mandatory for the testing of notifiable diseases in Germany), while three are fully commercialized but not yet licensed in Germany. Our results highlight a variability in the performance of different ELISA kits, which underscores the necessity of careful kit selection and interpretation of serological data, especially data derived from wild boar field samples.

We observed that all ELISA kits included in this study performed well with samples of domestic pigs, achieving both high sensitivity and specificity (>95% Sp and Se). This is consistent with previous reports of good performance of ELISA kits for detecting ASFV antibodies in domestic pigs [10]. However, in our study and previous reports, performance of ELISA kits varied significantly when wild boar samples were used. In particular, those of suboptimal quality due to varying stages of hemolysis and decay were challenging for measurements. This finding aligns with previously reported results, indicating that sample quality can significantly impact ELISA results [10]. In general, sensitivity was mainly moderate (45 to 70%), and specificity was fair to good (83 to 100%) when used on a larger cohort of wild boar sera. Of note, unlike routine confirmatory testing, where mainly ELISA-reactive sera are forwarded to the NRL, regional laboratories also submitted ELISA-negative wild boar samples specifically for this study, which reduced pre-selection towards positive cases and allowed a more balanced assessment of diagnostic performance. It has to be noted that we did not include qPCR results in our assessment. This would impact the overall diagnostic conclusion as some weak antibody-positive sera would have given a positive result in qPCR.

The pigtype ASFV Ab kit and the AsurDx ASFV AB Test kit demonstrated the highest sensitivity and specificity for wild boar samples in our dataset. However, these results have to be interpreted with particular caution, since only 91 wild boar samples were available for testing with the not-yet-licensed assays, compared to 310 samples assessed with licensed kits. Smaller sample size and potentially lower variability in this subset may inflate apparent performance and underestimate true error rates, especially for challenging field samples. As described previously, higher sample numbers, especially of challenging samples, will increase the error rate of any test [11,12]. Unfortunately, no further field samples could be acquired from affected German Federal States at that time. Thus, while our data suggest that these assays are promising candidates for use in wild boar surveillance, the current study cannot conclusively demonstrate that these kits would outperform the licensed kits in a broader field setting. Therefore, further evaluations on larger and more diverse wild boar cohorts are necessary. Although demonstrating high specificity, the ID Screen ASF Indirect kit showed lower sensitivity for wild boar samples and could lead to significant underestimation of seroprevalence rates. This finding contradicts previous studies that have reported good sensitivity of this kit when wild boar samples were used. The discrepancy may be attributed to differences in sample quality or testing workflows. Additionally, due to the different antigen specificities of the kits used in this study, data interpretation should be executed cautiously. In addition to sample quality and test format (indirect versus competitive ELISA), the antigen specificity of a given kit could influence its performance at different stages of ASFV infection. ASFV p30 is an early, highly immunogenic structural protein, expressed abundantly soon after infection and throughout the replication cycle, and is therefore often used as sensitive target for early antibody detection [8,13,14]. Antibodies against p30 can be detected from around 8–12 days post infection and typically rise rapidly thereafter [8,13]. By contrast, p72 is the major capsid protein expressed later in the replication cycle, and p72-specific antibody responses tend to appear slightly later but are generally robust and sustained during the following phases [8]. Assays that combine several structural proteins (e.g., p32/p72) may therefore provide broader coverage across different stages, but the relative contribution of each antigen to diagnostic sensitivity and specificity is difficult to pinpoint. In our study, kits targeting p72 alone (Ingezim PPA COMPAC) or in combination with other antigens (e.g., p54/p72, p32/p62/p72) showed moderate-to-good sensitivity for wild boar, whereas the p30-based and multi-antigen indirect assays generally performed best overall in this challenging matrix [13]. These findings are compatible with the observation that p30-focused assays are advantageous for detecting early or low-titer responses, while p72-based assays may preferentially capture animals in later infection or recovery, particularly when sample quality is not severely compromised. However, because the wild boar samples in this study originated from routine surveillance without precise information on time post infection, we could not formally stratify performance by infection stage, and conclusions must remain cautious.

Our multisite testing revealed that kit performance can also be influenced by slight differences in diagnostic workflows, e.g., if certain steps are executed manually or automated. This highlights the importance of fully standardized protocols for accurate and reproducible results. Interestingly, the LAVES laboratory, located in an ASF-free region, generated a false-positive result with the ID Screen ASF Indirect kit. This highlights the necessity of careful interpretation and confirmation (e.g., by the NRL and using techniques such as IPT) of serological data, especially in areas where ASF is not endemic. One possible, but yet unproven, explanation for the overall higher S/P% and the single false-positive result at LAVES is the use of a fully automated platform for ELISA processing at this site, which may differ in subtle ways (e.g., plate handling, washing, sealing/unsealing) from the predominantly manual workflows used in other laboratories. Yet, this platform was also used for all other tests and no sound explanation could be found in follow-up discussions.

The decision of which ELISA kit is most suitable to assess the ASFV-specific serological status of wild boar should be guided by the certain needs and context of the surveillance program. For areas where field samples of wild boar are impaired in quality, kits with higher sensitivity and tolerance to sample quality issues should be preferred. However, our study highlights that all wild boar field samples should be interpreted cautiously, considering the potential for and the consequences of false-positive and false-negative results. In particular, a validation of obtained ELISA+ results by IPT is recommended. Furthermore, combining serological testing with other diagnostic methods, such as PCR, can provide a more comprehensive picture of disease status and ASF dynamics in the field. It is of note that comprehensive comparison studies of qPCR kits and established qPCR methods could already facilitate the accuracy of diagnostic testing for ASFV [15,16]. Accurate serological data can contribute to improved risk assessments by providing reliable information on disease prevalence and distribution in wild boar populations. This can aid targeted surveillance and control measures, not only for ASF but also for other notifiable animal diseases, e.g., foot-and-mouth disease (FMD [17]) or classical swine fever (CSF [18]). Furthermore, continuous monitoring of ELISA performance and sample quality is crucial to ensure the accuracy and reliability of surveillance data.

Lastly, this study has limitations that should be acknowledged. For example, wild boar field samples tested in this study originated from animals that tested positive or at least questionable in serological testing at regional laboratories. This possibly introduced a sample bias towards positive results and may not fully reflect the performance of the kits in an unbiased cohort. However, by evaluating the performance of all accredited kits at the regional laboratories, we aimed to validate our findings with wild boar samples where the bias of testing only previously positive/questionable samples is minimal.

## 5. Conclusions

This study provides valuable insights into the performance of seven ELISA kits for detecting ASFV antibodies in domestic pigs and wild boar. Four of these kits are licensed in Germany under the respective Animal Health Law; all are commercially available. The performance of all kits tested was significantly hampered by severe hemolysis or advanced decomposition of field samples. Notably, the performance estimates for the not-yet-licensed kits, particularly for the wild boar cohort, are based on a markedly smaller sample set than for the licensed assays and should therefore interpreted carefully. However, the overall rate of correct interpretation was mainly satisfactory in all kits tested and best when using the Ingezim PPA COMPAC kit. Our findings highlight the importance of careful kit selection, sample quality, and data interpretation for effective ASFV surveillance and control. By keeping a high level of harmonization between regional and national laboratories, confirmation testing of positive/questionable results at the NRL will ultimately become even more comparable.

## Figures and Tables

**Table 1:** Number of samples from wild boar and domestic pig used in this study (tested at FLI).

	Manufacturer	Antigen	Wild Boar		Domestic Pig	
Total	—	—	425		272	
			Field = 400	Experiment = 5	Field = 92	Experiment = 180
**ID Screen ASF Indirect**	ID.vet	p32, p62, p72	310		229	
**ID Screen ASF Competition**	ID.vet	p32	310		229	
**Ingezim ASFV-R**	Gold Standard Diagnostics	cp213, p30	310		229	
**Ingezim PPA COMPAC**	Gold Standard Diagnostics	p72	310		229	
**pigtype ASFV Ab**	Indical	n/s	91		183	
**AsurDx ASFV Ab Test**	BioStone	p54, p72	91		183	
**ASFV Serum Ab indirect ELISA**	Tetracore	p30	91		183	

Legend: n/s = not specified.

**Table 2:** Number of field samples from wild boar used in this study (tested at regional authorities).

	Wild Boar	Comparative Panel
Total	693	46
**LAVES (Lower Saxony)**	285	46
**LLBB (Berlin-Brandenburg)**	40	46
**LUA (Saxony)**	368	46

**Table 3:** ELISA results and classification based on IPT results of all samples tested at the FLI.

	Wild Boar					Domestic Pig				
	TN	TP	FN	FP	Q	TN	TP	FN	FP	Q
**1. ID Screen ASF Indirect**	157	59	71	0	21	125	99	2	0	2
**2. ID Screen ASF Competition**	141	95	41	15	17	123	103	1	0	1
**3. Ingezim ASFV-R**	68	86	52	8	6	124	97	2	0	0
**4. Ingezim PPA COMPAC**	64	76	53	13	17	124	88	1	0	0
**5. pigtype ASFV Ab**	48	32	6	3	—	90	93	1	0	—
**6. AsurDx ASFV Ab Test**	46	27	3	2	4	88	93	1	0	2
**7. ASFV Serum Ab indirect ELISA**	49	24	5	3	0	89	92	2	1	0

Legend: TN = true negative, TP = true positive, FN = false negative, FP = false positive, Q = questionable.

## Data Availability

All raw data is accessible upon request to the corresponding author.

## Abbreviations

The following abbreviations are used in this manuscript:

ASF	African swine fever
ASFV	African swine fever virus
ELISA	Enzyme-linked immunosorbent assay
FLI	Friedrich-Loeffler-Institut
FN	False negative (result)
FP	False positive (result)
IPT	Immunoperoxidase test
LAVES	Niedersächsisches Landesamt für Verbraucherschutz und Lebensmittelsicherheit (Oldenburg)
LLBB	Landeslabor Berlin-Brandenburg (Frankfurt/Oder)
LUA	Landesuntersuchungsamt (Dresden)
NRL	National Reference Laboratory
TN	True negative (result)
TP	True positive (result)
